# Mr. Ring, in Reply to Mr. Davies

**Published:** 1808-01

**Authors:** John Ring

**Affiliations:** New-street, Hanover-square


### *To the Editors of the Medical and Physical Journal.*

GENTLEMEN,

YOUR correspondent, Mr. Davies, has so far followed the advice of the poet, in the line which he adopts as his motto, that if he communicates no instruction, he at least furnishes us with a considerable degree of entertainment. It is, however, a little remarkable, that any one who calls himself a medical practitioner, and pretends to the character of a man of science, should imagine Dr. Kentish's treatment of burns and scalds, and Dr. Kinglake's treat-

 ment ment of the gout, to be new discoveries, or new theories in medicine.

He thinks the difficulty of deciding these questions, is a reason why they should be suffered to rest; I think, on the contrary, with many of your other correspondents, that it is a reason why they should be the more discussed. I was lately consulted about another case, in a servant of a gentleman in Mansfield-street, in which the turpentine caused a very considerable erysipelatous inflammation far beyond the boundaries of the scald; which soon yielded to the following. ℞. Ammon. Muriat. ʒij. Acet. Distill. ℔ß. Aquæ ℔. jß. M.—I am also informed from good authority, that a medical practitioner, of the neighbourhood of Hanover-square, is now at Bath, in order to cure himself, if possible, of the ill effects of Dr. Kinglake's cooling plan; but hitherto without much benefit.

In the former instance of ill success from turpentine, which I communicated in this Journal, the patient confesses her skin to be irritable; but in the present, nothing of that kind had ever been observed till now; which renders it impossible always to determine when the stimulant applications will agree. I am therefore of opinion, that in all cases of this kind, if they are tried at all, and the inflammation does not soon subside, they should be exchanged for those of a sedative kind.

The other point to which Mr. Davies alludes is the Vaccine Contest, which has now lasted as long as the Trojan war; and Mr. Davies just begins to discover, that when he writes on this subject, he treads on tender ground.—*Sero sapiunt Phryges.*

He observes that I am a neighbouring practitioner; this he knew when he wrote his reflections on my conduct, and made his attack; it is, indeed, probable, that if we had lived farther apart, we should have been better neighbours.

He acknowledges that the cause of vaccination is much indebted to my humble endeavours; but still seems unwilling to admit, that it is justifiable to refute the misrepresentations, or correct the errors of an author, unless you have an opportunity of doing it in his life-time. Were this sort of reasoning once allowed, the antivaccinists would gain a complete victory; for they publish their false reports much faster than it is possible to refute them.

He says, if my improvement of the well-known adage ' consists only in recapitulating their errors and prejudices, and holding up their memory to obloquy and contempt, when when the individuals themselves are beyond the reach of self-defence,' it reflects, in his opinion, but little credit upon the living. In this respect, I most cordially agree with him; and have not the least doubt, most of the readers of what I have written will agree with me, that Mr. Davies has here thrown out an illiberal and unjust insinuation. As to the *imaginary* mistakes of the antivaccinists, which he talks about, I leave it to him to discover those imperfections; but as to their *real* mistakes, as they affect the characters of the living, and the welfare and happiness of the present age and posterity, it is our duty to detect them. As far as regards their private characters, it may, indeed, be the duty of those who are "liable to other imperfections," to draw a veil over them, and to bury them in the same grave with their authors; but when a publication survives its author, the fallacies of that publication ought to be pointed out.

Mr. Davies says, I insinuate that he classes 'new theories in medicine with the vaccine practice, as if incapable of distinguishing between theory and practice.'—He then makes himself witty at my expence; and tells his readers, that as soon as my *favourite* subject was only hinted at, my thoughts were so absorbed in it, that I overlooked all which was mentioned in the former part of his paper: otherwise he thinks a Davus might have understood it as well as an Œdipus.

Never was there a more gross and palpable misrepresentation than this. Great wits have short memories. By turning to the said paper, in the Journal for September, it appears, that his words are as follows.

"We were given to understand, that after an appeal to the Royal College of Physicians, for their opinion on Vaccination, private communications on that subject would be for the most part suspended; but as several pages of the last Number of your Journal were occupied in treading the beaten path of animadversions on the Antivaccinists, you will allow me to make an observation or two on medical controversies in general, *and this in particular*.

"When *new theories in medicine* are discovered, however ingenious in themselves, they will be received by experienced practitioners with caution, and examined with candour; nor should the discoverers be too sanguine in their expectations of a general adoption of their systems, till a sufficient period of time has elapsed to confirm the success of the practice."

Here

Here it is evident, that I did *not* overlook all which was mentioned by Mr. Davies in the former part of his paper; that Mr. Davies *did* class new theories in medicine with the vaccine practice, as if incapable of distinguishing between theory and practice; and that he wishes his readers to believe, what it is scarcely possible he can believe himself, that I have insinuated, as he has done on another occasion, what has no foundation in truth.

He gives his opinion, that the Report of the Royal College of Physicians is candid and judicious; and then modestly congratulates himself on being found in such honourable company; where I should never have thought of placing him, not knowing any company where he would appear to greater disadvantage.

He professes to be a friend to the improvement of the healing art; and to have a particular partiality for the science, and a regard for the welfare of mankind. All this may be true; but he has been rather unfortunate in his way of shewing it. The College of Physicians, whose Report he acknowledges to be candid and judicious, passed a severe censure on all the books written against vaccination, the principal of which were those of Dr. Moseley, Dr. Rowley, and Mr. Birch; declaring, that in their opinion, the charges of its producing other diseases, are either the inventions of *designing*, or the mistakes of *ignorant* men; and that the *prints* representing such diseases, which have been published with a view to terrify weak and ignorant people, originate either in *gross ignorance*, or *wilful misrepresentation*.

Mr. Davies thinks vaccination is now reduced to regular and established practice; and that it is no longer necessary to maintain a contest on the subject; it is however to be lamented, that while too many of the respectable part of the faculty tamely look on, and, with an unaccountable supineness, see the small-pox commit its ravages, the dregs of the profession are straining every nerve to delude the public, and to disseminate this deadly contagion. The friends of vaccination, therefore, instead of relaxing in their endeavours, and retiring from the contest, as Mr. Davies so earnestly advises, for reasons best known to himself, ought to renew the combat with fresh vigour, and to redouble their exertions.

The small-pox is so far from being subdued, that it has lately raged with great violence, to the disgrace of those who ought to watch over the public health; and the enemies enemies of vaccination are still asserting the most impudent and abominable falsehoods. They pretend that some children fell victims to the cow-pock, who never had the disorder; and others who died of a different disease. They also pretend, that one of the friends of vaccination labours under a *noli me tangere*; but if they dare to make the experiment, they will find, that it is a *nemo me impune lacesset*.

Mr. Davies takes great pains to convince his readers, that he is not so inimical to useful communications as I represent him to be. This is a point, of which every one must judge for himself. He once more calls on the Editors for something new. I hope he will enable them to produce something new, and at the same time useful; —useful to *the public*, as well as to *the profession*.

As to the "other Commentator" on his letter, whom he alludes to, I no more know than he himself, whether the signature under which he appears is real or fictitious; but this I know, that he lays down rules for medical controversies, which neither he, nor any other man, should disdain to follow.

I am, &c.

JOHN RING.

*New-street, Hanover-square.*



